# Serum inflammatory factors are positively correlated with the production of specific antibodies in coronavirus disease 2019 patients

**DOI:** 10.1038/s41423-020-00551-1

**Published:** 2020-09-22

**Authors:** Meijuan Zheng, Yong Gao, Siyu Liu, Dandan Sun, Fan Yang, Lu Zong, Min Zhang, Zhigang Tian, Yuanhong Xu, Haoyu Sun

**Affiliations:** 1 Department of Clinical Laboratory, First Affiliated Hospital of Anhui Medical University, Hefei, Anhui China; 2 Department of Clinical Laboratory, Fuyang Second People’s Hospital, Fuyang, Anhui China; 3 Hefei National Laboratory for Physical Sciences at Microscale, the CAS Key Laboratory of Innate Immunity and Chronic Disease, School of Basic Medical Sciences, Division of Life Sciences and Medicine, University of Science and Technology of China, Hefei, China; 4 Institute of Immunology, University of Science and Technology of China, Hefei, China

**Keywords:** Prognostic markers, Humoral immunity

The ongoing spread of coronavirus disease 2019 (COVID-19) constitutes an international concern on an unprecedented scale. To date, over 23 million people have been diagnosed with COVID-19 worldwide, and this disease has caused more than 800,000 deaths. Hyperinflammation elicited by severe acute respiratory syndrome coronavirus 2 (SARS-CoV-2) has been reported to contribute to illness severity and death.^1,2^ Humoral immune responses play important roles in therapy and prophylaxis for SARS-CoV-2 infection. Since the receptor-binding domain (RBD) of the SARS-CoV-2 spike (S) glycoprotein binds to angiotensin-converting enzyme 2 to trigger virion endocytosis, antibodies against this domain may be able to neutralize SARS-CoV-2 and possibly provide protective immunity in COVID-19 patients.^3^ Clinical trials investigating the administration of convalescent plasma and the interleukin (IL)-6 antagonist tocilizumab to treat COVID-19 patients are currently underway,^4^ but the overly robust expansion of antibody-secreting cells (ASCs) could play a major role in the pathogenicity of SARS-CoV-2 in COVID-19 patients.^5^ Thus, a detailed characterization of the associations between humoral immune responses and inflammatory factors could result in a better understanding of SARS-CoV-2-host interactions in COVID-19 patients.

In the current study, the levels of RBD-specific IgG, RBD-specific IgA, and the frequencies of ASCs and ICOS+ T follicular helper (TFH) cells were found to be higher in severely affected COVID-19 patients than those in nonseverely affected patients. Follow-up analysis of COVID-19 patients demonstrated that humoral immune responses were positively correlated with the levels of IL-6, C-X-C motif chemokine 10 (CXCL10), and C5a. Positive correlations between the serum CXCL13 level and the levels of IL-6 and CXCL10 were also noted in COVID-19 patients. Taken together, these results indicate that there is a close relationship between humoral immunity and inflammatory factors, and the generation of protective humoral immunity could be a double-edged sword in COVID-19 patients.

A total of 54 hospital-admitted COVID-19 patients (41 nonsevere cases and 13 severe cases) were assessed in the present study. The clinical characteristics of the patients and results of their laboratory tests are summarized in Supplementary Tables 1 and 2. The levels of total anti-RBD antibodies in the severe patients were higher than those in the nonsevere patients and healthy controls from 5 to 33 days after the onset of illness. Furthermore, a comparison of humoral immune responses between the nonseverely and severely affected patients showed significantly higher levels of IgG and IgA in severe patients than in the nonsevere patients or healthy controls. Because ASCs are critical for the production of antibodies and TFH cells provide help for B cells to induce antibody responses after infection, the presence of CD24^−^CD38^+^CD19^+^ ASCs and ICOS^+^ TFH cells was investigated in COVID-19 patients. In line with the increased levels of anti-RBD antibodies, the severe patients also exhibited higher percentages of ASCs and ICOS^+^ TFH cells than did the nonsevere patients (Fig. 1a). Collectively, these results indicated that severe COVID-19 illness induced strong humoral immune responses, which is consistent with previous studies showing higher IgG titers in severe patients than in nonsevere patients.^6^

**Fig. 1:**
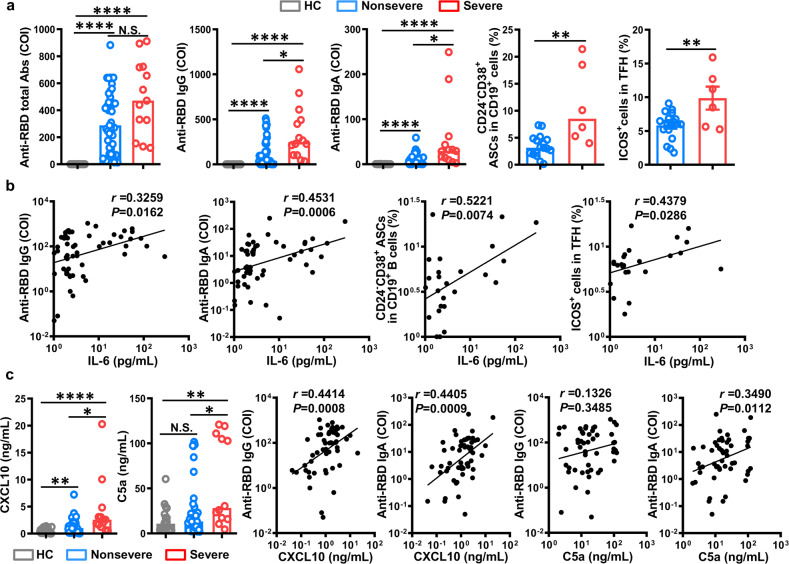
Associations between increased serum inflammatory factor levels and antibodies against severe acute respiratory syndrome coronavirus 2 (SARS-CoV-2) in coronavirus disease 2019 (COVID-19) patients. **a** Serum levels of total anti-receptor-binding domain (RBD) antibodies, anti-RBD IgG, anti-RBD IgA, CD24^−^CD38^+^ antibody-secreting cells (ASCs) gated on CD3^−^CD19^+^ lymphocytes, and ICOS^+^ cells gated on CD3^+^CD4^+^CXCR5^+^ follicular helper T (TFH) cells in healthy controls, nonseverely affected COVID-19 patients, and severely affected COVID-19 patients. **b** Correlations between the serum IL-6 level and the levels of anti-RBD IgG and anti-RBD IgA and the percentages of CD24^−^CD38^+^ ASCs and ICOS^+^ TFH cells in COVID-19 patients. **c** Serum levels of the inflammatory factors C-X-C motif chemokine 10 (CXCL10) and C5a in the healthy controls, nonseverely affected COVID-19 patients, and severely affected COVID-19 patients, and correlations between these two inflammatory factors and the serum levels of anti-RBD IgG and anti-RBD IgA in COVID-19 patients. Bar graphs show the median or mean ± SEM. The Mann–Whitney *U* test and Student’s *t*-tests were conducted for two-group comparisons, according to the data distribution, and one-way ANOVA was conducted for three-group comparisons. Nonparametric (Spearman) and parametric (Pearson’s) correlation analyses were conducted between variables. *p* < 0.05 was considered significant. *p < 0.05; **p < 0.01; ****p < 0.0001; N.S. not significant

Hyperinflammation is reported to be associated with disease severity and death in COVID-19 patients. In the current study, the average serum level of IL-6 was found to be significantly higher in the severe patients than in the nonsevere patients and healthy controls (Supplementary Fig. 1). As a proinflammatory cytokine, IL-6 has been shown to be involved in antibody production by B cells.^7^ To assess this function in COVID-19, the association between IL-6 and humoral responses was evaluated in COVID-19 patients. As shown in Fig. 1b, significant correlations were observed between the levels of IL-6 and humoral immunity parameters, including the levels of anti-RBD IgG, anti-RBD IgA, ACSs, and ICOS^+^ TFH cells.

Moreover, higher levels of CXCL10 and C5a were observed in the severe patients than in the nonsevere patients and healthy controls (Fig. 1c), which is consistent with previous reports on COVID-19.^8,9^ In an effort to further characterize the relationships between these two inflammatory factors and humoral immunity, the correlations between these two inflammatory factors and humoral responses were investigated in COVID-19 patients. The expression of CXCL10 was strongly correlated with the levels of anti-RBD IgG and IgA in COVID-19 patients, which is a feature also observed in patients with autoimmune diseases.^10^ C5a/C5aR1 interactions in CD4^+^ T cells are associated with an increased percentage of TFH cells and an elevated level of autoantibody production.^11^ Concordant with these results, we found a strong correlation between the levels of C5a and anti-RBD IgA; however, only a weak correlation was observed between the levels of C5a and anti-RBD IgG (Fig. 1c). In addition, as a ligand of CXCR5, CXCL13 was also found at a higher serum level in the severe COVID-19 patients than in the nonsevere patients. Positive correlations were observed between the levels of CXCL13 and the level of IL-6 or CXCL10; however, no significant relationship was noted between the CXCL13 and C5a levels (Supplementary Fig. 2). Collectively, these results suggest that humoral immune responses are associated with the inflammatory factors IL-6, CXCL10, and C5a in COVID-19 patients.

Our study showed that the severely affected patients displayed higher levels of anti-RBD antibodies, increased frequencies of ASCs and ICOS^+^ TFH cells, and elevated levels of CXCL13. Importantly, the elevated levels of serum IL-6, CXCL10, and C5a were correlated strongly with humoral immune responses, constituting further evidence of a close relationship between inflammatory factors and humoral immune responses in this context. It has been reported that antibody responses against viruses can lead to disease via antibody-dependent enhancement (ADE), which is characterized as antibody-mediated effects on viral entry but also harmful inflammatory responses.^12^ More notably, convalescent plasma from recovered COVID-19 patients with high levels of anti-SARS-CoV-2 antibodies has been used for the treatment of COVID-19 patients, but this treatment has at least a theoretical possibility of being associated with ADE^13^ and may therefore have the unintended consequence of enhancing the severity of COVID-19 infection. Given that the immunopathological effects of ADE elicited by nonneutralizing antibodies targeting non-RBD antigens have been described in the context of SARS infection,^14^ such immunopathological effects, not just a lack of protection, constitute a major concern with regards to assessing the effects of antibody-mediated enhancement on SARS-CoV-2 infection.

Collectively, the results reported to date indicate that additional studies are required to ascertain whether biomarkers that reflect associations between humoral responses and inflammatory factors can be used to predict COVID-19 severity. The limited number of severely affected patients and the absence of neutralizing antibody measurements somewhat limited our study. Effective control of SARS-CoV-2 requires further investigation of the mechanism underlying the correlations between humoral immunity and inflammatory factors in severe COVID-19, and the results of such studies could be used to guide immunotherapy with passive antibodies while controlling hyperinflammation.

## Supplementary information

Supplementary Material-20200901-2

Supplementary Figure 1

Supplementary Figure 2
