# Supplementary material for: Serum inflammatory factors are positively correlated with the production of specific antibodies in coronavirus disease 2019 patients
Source: Cell Mol Immunol. 2020 Sep 22;17(11):1180–2. doi: 10.1038/s41423-020-00551-1 (PMC7506822; doi:10.1038/s41423-020-00551-1)
Supplement: Supplementary file 1 — Supplementary Material-20200901-2 [file 41423_2020_551_MOESM1_ESM.docx]

**Supplementary Information**

**Materials and Methods:**

**Patients**

This study included a total of 54 cases of COVID-19. All enrolled subjects were diagnosed as being infected with SARS-CoV-2 at the First Affiliated Hospital of Anhui Medical University (Hefei, China) and Fuyang Second People’s Hospital (Fuyang, China). For all recruited subjects, peripheral-blood testing results during their stays in the hospital, and their detailed information are summarized in the Supplementary Tables 1 and 2. This study was approved by the Ethics Committee of First Affiliated Hospital of Anhui Medical University.

**Flow cytometry**

Isolated lymphocytes from blood were resuspended and stained with mouse anti-human monoclonal antibodies. Data were collected using a BD FACSCanto Plus flow cytometer (BD Biosciences, San Diego, CA) and analyzed with FlowJo VX (Tree Star, Inc., Ashland, OR). The antibodies used were FITC–anti-CXCR5, PE-anti-CD24, PerCP-anti-CD45, APC-anti-CD38, PE-CY7-anti-CD19, PE-CY7-anti-CD4, APC-CY7-anti-CD8, BV421-anti-ICOS, and BV510-anti-CD3 (all from BD Biosciences).

**Cytometric bead array**

Inflammatory cytokines (IL-6) in serum were detected using the BD Cytometric Bead Array (CBA) Human Inflammatory Cytokines kit (BD Biosciences), and chemokines (CXCL-10) were analyzed using the BD Cytometric Bead Array (CBA) Human Chemokine kit (BD Biosciences). IL-6 and CXCL10 levels were measured according to the manufacturer’s instructions. Briefly, sera (50 μL) from SARS-CoV-2-infected patients and healthy donors were first incubated with mixed capture beads (50 μL), to which 50 μL of PE Detection Reagent were then added. After being washed with buffer, the samples resuspended in 300 μL of buffer were analyzed using a BD FACS Calibur flow cytometer.

**Enzyme-linked immunosorbent assay**

C5a concentration was analyzed with a RayBio Human C5a Enzyme-linked immunosorbent assay (ELISA) kit (ELH-CCC5a, RayBiotech, Norcross, GA). In brief, all reagents, samples and standards were first prepared according to the instructions. Then a volume of 100 μL of each standard and each sample was incubated for 2.5 h. Each of these mixtures was then incubated with a volume of 100 μL of prepared biotin antibody for 1 h. Each of the resulting mixtures was then incubated with 100 μL of prepared streptavidin solution for 45 minutes. And each of these resulting mixtures was incubated with TMB One-Step Substrate Reagent for 30 minutes, after which Stop Solution was added. The absorbance of each final mixture at a wavelength of 450 nm was immediately measured. All procedures were performed at room temperature.

CXCL13 concentration was measured with Human CXCL13 SimpleStep ELISA Kit (BCA-1) (ab269370, Abcam, Cambridge, MA). Briefly, add 50 µL of all prepared samples or standard to corresponding wells of 96-well plates. Followingly, 50 µL antibody cocktail were added and incubated for 1 h. After washing wells three times with 350 µL Wash Buffer PT, 100 µL TMB Development Solution were added and incubated for 10 minutes in dark. Then added 100 µL Stop Solution and read OD at 450nm. All procedures were performed at room temperature.

**Chemiluminescence microparticle immunoassay**

Total antibody, IgG antibody and IgA antibody against the RBD domain of the spike protein of SARS-CoV-2 were evaluated using chemiluminescence microparticle immunoassay (CMIA) kits (Xiamen Wantai Biological Pharmacy Enterprise, China). RBD-specific total antibody was detected based on the double-antigen sandwich principle. RBD-specific IgA and IgG antibodies were detected using indirect principles. The levels of anti-RBD specific total antibody, IgG and IgA were measured according to the manufacturer’s instructions. The Carris 200 calculates cut off index (COI). COI values <1.0 are considered negative; otherwise they are considered positive.

**Statistical analysis**

The signiﬁcance of differences was analyzed using Graphpad Prism 8.0. Bar graphs show the median or mean ± SEM. The Mann-Whitney *U* test and Student’s *t*-tests were conducted for two-group comparison, with one-way ANOVA for three-group comparison according to data distribution. Nonparametric (Spearman) and parametric (Pearson’s) correlation analyses were conducted between variables. *p* < 0.05 was considered significant. **p* < 0.05, ***p* < 0.01, ****p* < 0.001, *****p* < 0.0001, N.S., not significant.

**Supplementary Figure legends**

**Supplementary Figure 1:** Serum IL-6 levels in healthy controls, nonseverely affected and severely affected COVID-19 patients. Bar graphs show the median values. One-way ANOVA was conducted for three-group comparison according to data distribution. *p* < 0.05 was considered significant. ****p* < 0.001, *****p* < 0.0001.

**Supplementary Figure 2:** Serum CXCL13 levels in nonseverely affected and severely affected COVID-19 patients, and the relationship between these levels and the levels of IL-6, CXCL10 and C5a. **a.** Serum levels of CXCL13 in nonsevere and severe COVID-19 patients. **b.** Correlations between the serum CXCL13 level and the levels of IL-6, CXCL10 and C5a in COVID-19 patients. Bar graphs show the median values. The Mann-Whitney *U* test was used for two-group comparison. Nonparametric (Spearman) correlation analyse was conducted between variables. *p* < 0.05 was considered significant. ***p* < 0.01.

**Table 1 Characteristics of patients infected with SARS-CoV-2**

|  | All patients  （n=54） | Nonsevere  （n=41） | Severe  （n=13） |
| --- | --- | --- | --- |
| Age  Sex  Male  Female  Days after illness onset  Before treatment nucleic acid test  Comorbidities  Hypertension  Cardiovascular disease  Diabetes  COPD  Chronic liver disease | 48.31(15.17)  38/54(70.37%)  16/54(29.63%)  20.91(7.94)  54/54  11(20.37%)  4(7.41%)  9(16.67%)  2(3.70%)  3(5.56%) | 43.88(13.04)  26/41(63.41%)  15/41(36.59%)  21.39(8.35)  41/41  8(19.51%)  2(4.88%)  3(7.32%)  0  2(4.88%) | 62.31(13.05)  12/13(92.31%)  1/13(7.69%)  19.38(6.55)  13/13  3(23.08%)  2(15.38%)  6(46.15%)  2(15.38%)  1(7.69%) |

**Table 2 Clinical laboratory tests of patients infected with SARS-CoV**

|  | | All patients  （n=54） | Nonsevere  （n=41） | | | Severe  （n=13） | P value | |
| --- | --- | --- | --- | --- | --- | --- | --- | --- |
| White blood cell, × 10⁹/L  Neutrophil, × 10⁹/L  Lymphocyte, × 10⁹/L  B cell, cells/μL  CD4^+^ T cell, cells/μL  Haemoglobin, g/L  Platelet count, × 10⁹/L  Albumin, g/L  Alanine aminotransferase, U/L  Aspartate aminotransferase, U/L  Total bilirubin, mmol/L  Potassium, mmol/L  Sodium, mmol/L  Creatinine, μmol/L  Lactate dehydrogenase, U/L  D-dimer, mg/L  C-reactive protein, mg/L | 6.202(2.669)  3.999(2.467)  1.559(0.640)  143.704(67.582)  493.200（218.356)  128.274(18.164)  207.667(69.147)  40.059(5.960)  36.726(28.340)  26.720(22.097)  11.928(5.720)  4.122(0.409)  139.902(2.997)  67.930(33.836)  206.241(73.966)  1.201(1.895)  13.635(33.547) | | | 6.114(1.879)  3.754(1.514)  1.713(0.610)  155.895(70.737)  515.035(224.343)  129.629(17.423)  207.122(58.755)  41.190(6.187)  31.566(21.246)  25.071(24.301)  10.946(5.234)  4.183(0.356)  139.763(2.515)  65.846(17.109)  181.951(42.800)  0.722(1.092)  6.995(13.999) | 6.482(4.425)  4.772(4.290)  1.072(0.484)  121.353(57.507)  435.633(199.993)  124.000(20.474)  209.385(98.010)  36.492(3.335)  53.000(40.800)  31.923(12.230)  15.023(6.287)  3.933(0.510)  140.339(4.280)  74.500(63.396)  282.846(98.406)  2.712(2.937)  34.575(60.759) | | | 0.2762  0.6921  0.0011  0.1574  0.3106  0.3350  0.5383  <0.0001  0.1026  0.0080  0.0237  0.0543  0.5517  0.6063  <0.0001  0.0012  0.3748 |
