# Supplementary figures and images for: Serum inflammatory factors are positively correlated with the production of specific antibodies in coronavirus disease 2019 patients
Source: Cell Mol Immunol. 2020 Sep 22;17(11):1180–2. doi: 10.1038/s41423-020-00551-1 (PMC7506822; doi:10.1038/s41423-020-00551-1)

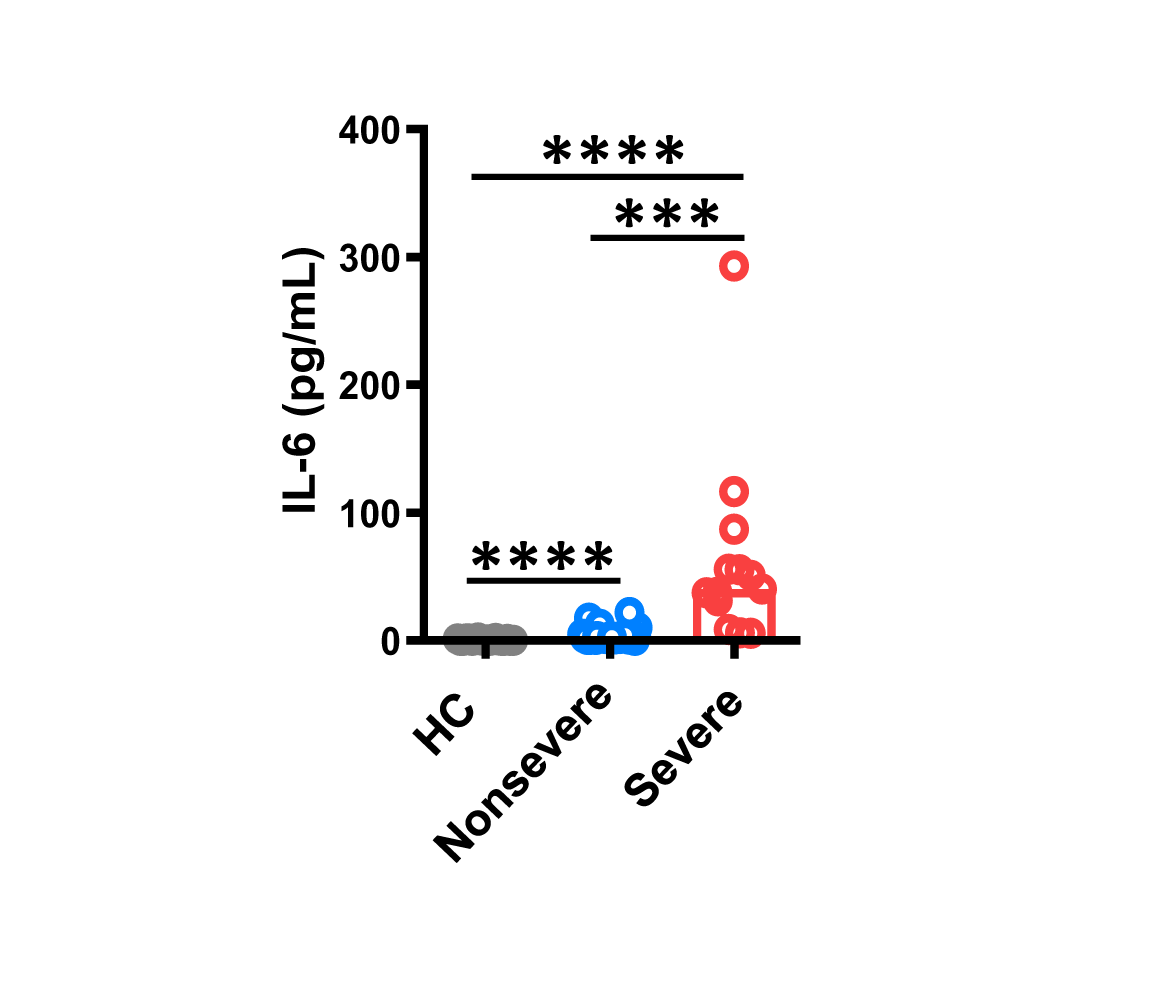

Supplement: Supplementary file 2 — Supplementary Figure 1 [file 41423_2020_551_MOESM2_ESM.tif]

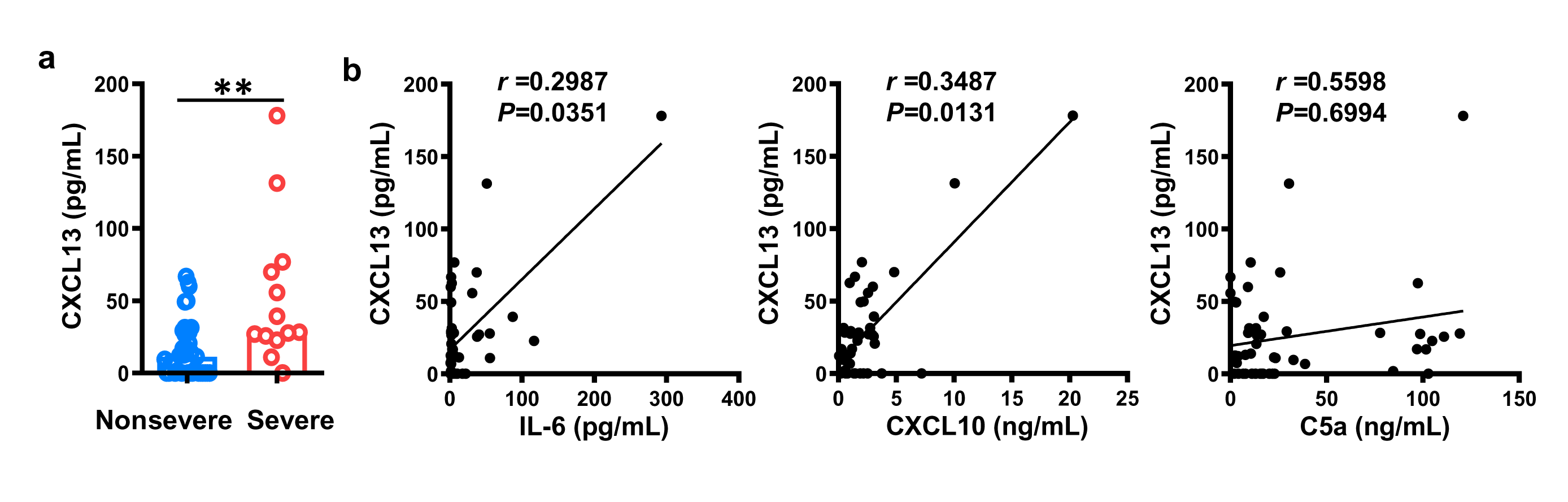

Supplement: Supplementary file 3 — Supplementary Figure 2 [file 41423_2020_551_MOESM3_ESM.tif]
